# Expression, Purification, and Functional Characterization of Atypical Xenocin, Its Immunity Protein, and Their Domains from *Xenorhabdus nematophila*

**DOI:** 10.1155/2013/746862

**Published:** 2013-12-18

**Authors:** Jitendra Singh Rathore

**Affiliations:** School of Biotechnology, Gautam Buddha University, Yamuna Expressway, Greater Noida, Uttar Pradesh 201308, India

## Abstract

*Xenorhabdus nematophila, *a gram-negative bacterium belonging to the family Enterobacteriaceae is a natural symbiont of a soil nematode from the family Steinernematidae. In this study cloning, expression, and purification of broad range iron regulated multidomain bacteriocin called xenocin from *X. nematophila* (66 kDa, encoded by *xcinA* gene) and its multidomain immunity protein (42 kDa, encoded by *ximB* gene) have been done. *xcinA-ximB* (N′ terminal 270 bp), translocation, and translocation-receptor domain of *xcinA*, *ximB*, and its hemolysin domain were cloned, expressed, and purified by single step Ni-NTA chromatography under native conditions. In the functional characterization, neutralization of *xcinA* toxicity by immunity domain of *ximB* gene was determined by endogenous assay. Exogenous toxic assays results showed that only the purified recombinant xenocin-immunity domain (10 kDa) protein complex had toxic activity. Atypical cognate immunity protein (42 kDa) of xenocin was fusion of immunity domain (10 kDa) and hemolysin domain (32 kDa). *In silico* analysis of immunity protein revealed its similarity with hemolysin and purine NTPase like proteins. Hemolytic activity was not observed in immunity protein or in its various domains; however, full-length immunity protein lacking Walker motif showed ATPase activity. Finally, using circular dichroism performed secondary structural analyses of all the recombinant proteins/protein complexes.

## 1. Introduction

Bacteriocins are toxins produced by the bacteria to inhibit the growth of similar or closely related bacterial strain(s) during stress conditions [1]. They are structurally, functionally, and ecologically diverse, produced by almost all major lineages of Eubacteria and Archaebacteria [2]. Ribosomal encoded bacteriocins are generally secreted in the extracellular milieu by the producers where they recognize specific receptors on the surface of susceptible or target cells. They induce toxicity in the target cells by different mechanisms like enzymatic nuclease (DNase or RNase) or pore formation in cytoplasmic membrane [3]. Their structure comprises of three distinct domain organizations: (i) a domain involved in recognition of specific receptor R, (ii) a domain involved in translocation T, and (iii) a domain responsible for their toxic activity C. Molecular mass of ribosomal encoded bacteriocins vary from ~25 to 80 kDa and are broadly classified into two groups, group A and B, based on their cross-resistance [4]. These proteins have received increasing attention due to their potential use as preservatives in the food industry or in the therapeutic applications for clinical usage [5].

*Xenorhabdus nematophila *is a motile gram-negative bacterium belonging to the family Enterobacteriaceae, and forms a symbiotic association in the gut of soil nematode from the family Steinernematidae [6, 7]. Free-living forms of the bacterium have not yet been isolated from soil or water sources, which suggests that the symbiotic association may be essential for the survival of the bacteria in the environment. Nematodes enter the insect hemocoel via different routes. Infective juvenile (IJs) enters digestive tract of the insect larva and subsequently penetrates into hemocoel of the host insect. The nematode can also gain access to the hemocoel via the respiratory spiracles or by penetrating directly through insect cuticle [8]. Bacteria, in turn, are essential for effective killing of the insect host and are required by the nematode to complete its life cycle [8, 9]. *X. nematophila* can be grown under standard laboratory conditions. Growth *in vitro* is probably supported by the rich nutrient supply of the laboratory growth media and lack of competition that normally exists in the soil environment. As the bacteria enter stationary phase of growth cycle, they secrete several extracellular products, which include lipase(s), phospholipase(s), and protease(s), and several broad spectrum antibiotics that can be assayed in the culture media [10, 11]. These extracellular products are believed to be secreted in the insect hemolymph when the bacteria enter stationary phase. These degradative enzymes break the macromolecules from insect cadaver to provide the developing nematode with nutrient supply, while the antibiotics suppress contamination of the cadaver by other microorganisms. Cytoplasmic inclusion bodies, composed of highly expressed crystalline proteins, are also produced by the bacterium during stationary-phase growth [12]. In our earlier study we have identified iron regulated bacteriocin from *X. nematophila* known as xenocin [13]. Recombinant xenocin-immunity protein complex is toxic to six bacterial genus like *Bacillus, Enterobacter, Enterococcus, Citrobacter, Serratia, and Stenotrophomonas* [13]. Xenocin-immunity protein complex has atypical features which include the following. (1) Tol box which has been replaced by Ton box from N′ terminal end of translocation domain of xenocin [13]. (2) There is only 30% similarity of xenocin with other bacteriocins [13]. (3) Size of its cognate immunity protein is 42 kDa, whereas 10–16 kDa have been reported in other prokaryotic systems [4]. Immunity protein of *X. nematophila* is a fusion of two different domains, immunity domain and hemolysin domain. (4) Immunity protein has ATPase activity, although Walker motif is missing in its primary amino acid sequence. (5) Xenocin-immunity protein complex is secretory in nature without any signal sequence.

In this study we have cloned, expressed, and purified all the possible domains of *xcinA* and *ximB *genes. Neutralization of *xcinA* toxicity by immunity protein domain is determined by endogenous assay. Exogenous toxicity assays were performed with purified recombinant xenocin-immunity domain protein complex and other domains. *In silico* study of the immunity protein showed its similarity with hemolysin and purine NTPase like protein; therefore, hemolysis and ATPase assays were performed. Finally, secondary structural analysis of recombinant xenocin-immunity domain protein complex, catalytic-immunity domain protein complex, immunity protein, and its hemolysin domains were performed with circular dichroism.

## 2. Material and Methods

### 2.1. Bacterial Strain, Media, and Culture Conditions

All the chemicals were purchased from Sigma (Sigma-Aldrich) except where otherwise mentioned. Ligase and endonucleases were purchased from Promega (Madison, USA). Vector pGEM-T easy was procured from Promega (Madison, USA). Vector pQE30, Ni-NTA agarose resin and QIA quick spin columns were from Qiagen (Germany). Oligonucleotides were custom synthesized by Sigma. *E. coli *strains DH5*α* (Bethesda Research Laboratories) were used as the host for cloning. *E. coli* BL 21(DE3) pLysS strain from Novagen and M 15 strain from Qiagen were used in the expression studies. The plasmid vector pGEM-T Easy from Promega (Madison, USA) were used for PCR cloning. LB medium was used for growing bacterial strains. Ampicillin, Kanamycin, and Chloramphenicol were used in the concentration of 100, 35, and 25 *μ*g mL^−1^, respectively.

### 2.2. Phylogenetic Analysis

Phylogenetic analysis of immunity protein was done by a method as described earlier [14]. Briefly, amino acids of all the protein sequences that matched with immunity protein were aligned by CLUSTALW (Mac vector 7.0), and a tree was constructed using neighbour-joining method, with the best tree mode in the Mac vector version 7.0 (Oxford Molecular, England) program.

### 2.3. Cloning

#### 2.3.1. Xenocin-Immunity Domain

**Figure 1:**
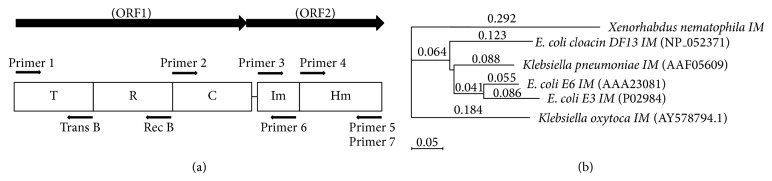
(a) Domain map of xenocin and immunity protein. T: translocation domain; R: receptor domain; C: catalytic domain of xenocin; Im: immunity domain; Hm: hemolysin domain of immunity protein. Primers position and orientation for cloning of different domains depicted with arrows. (b) Phylogenetic analysis of immunity protein from *X. nematophila* with similar protein sequences from other bacterial species. *E. coli cloacin* DF13 (NP_052371); *Klebsiella pneumonia* (AAF05609); *E. coli* colicin-E6 (AAA23081); *E. coli* E3 (P02984); *Klebsiella oxytoca* (AY578794.1).

Primer pairs used for cloning studies are shown in Figure 1(a). All the constructs were amplified from the 4.3 kb genomic DNA fragment of *X. nematophila*. The ORF 1 encoding *xcinA *gene and partial ORF 2 encoding immunity domain of *ximB* gene were obtained by PCR amplification using primer 1 with a *BamH*I site at the 5′ end and a reverse primer 6 with *Hind*III site at the 3′ end. The amplified product (2 kb) was ligated in pGEM-T Easy and pQE30 vector, producing pJC5 and pJC6 plasmids, respectively.

#### 2.3.2. Catalytic-Immunity Domain

The catalytic domain (318 bp) of *xcinA* gene was cloned with the immunity domain (first 270 bp of *ximB* gene) of *ximB* gene using primer pair 2 and 6 as described earlier [13].

#### 2.3.3. Translocation Domain of Xenocin

Primer 1 with a *BamH*I site at the 5′ end and a reverse primer TransB with *Nco*I site at the 3′ end were used to amplify the translocation domain of *xcinA* gene. Amplified product of 1 kb was ligated in pGEM-T Easy vector and pQE30 vector, producing pJC7 and pJC8 plasmids, respectively.

#### 2.3.4. Translocation-Receptor Domain of Xenocin

Primer 1 with a *BamH*I site at the 5′ end and a reverse primer RecB with *Hind*III site at the 3′ end were used to amplify the translocation-receptor domain of *xcinA* gene. The amplified product (1.5 kb) was ligated in pGEM-T Easy vector and pQE30 vector producing pJC9 and pJC10 plasmids, respectively.

#### 2.3.5. Immunity and Its Hemolysin Domain

Primer 3 with *BamH*I site at the 5′ end and a reverse primer 5 with *Hind*III site at the 3′ end used for *ximB* cloning and forward primer 4 without any restriction site and backward primer 7 without any restriction site were used for cloning *ximB* hemolysin domain. The amplified products of 1 kb and 700 bp were ligated in pGEM-T Easy vector producing pJC11 and pJC12 plasmids. The 1 kb amplified product was further ligated in pQE30 and 700 bp amplified product was ligated in pQE31 expression vector producing pJC13 and pJC14 plasmids, respectively.

#### 2.3.6. Cloning of *XcinA-XimB* (255 bp) Gene(s) under Native Promoter

A 2.330 kb DNA fragment containing both *xcinA* and immunity domain of *ximB* gene with native promoters was amplified using XenocinF1 (300 bp upstream of start codon of *xcinA* locus) and primer 6 and cloned in pGEM-T Easy vector producing pXIM construct. The empty pGEM-T Easy vector designated as pGEM was used as control. Both plasmids were transformed in DH5*α* cells. The XIM and GEM strains were used for endogenous assays. All the constructs and strains are listed in Table 1.

### 2.4. Expression and Purification

The plasmids pJC6, pJC8, pJC10, pJC13, and pJC14 were transformed in M15 cells where as pJC4 was transformed in* E. coli* BL 21(DE3) pLysS cells. The resulting strains JC4, JC6, JC8, JC10, JC13, and JC14 were used for expression and purification of recombinant proteins under the control of IPTG inducible T7 promoter as per the protocol described earlier [13]. Briefly, overnight grown cultures were diluted 100 fold in fresh 50 mL LB medium and grown till the OD_600_ reached 0.5. Culture was induced by adding 1 mM final concentration of IPTG and incubated at 30°C for 6 hours. Cells were harvested and washed with 40 mL of cold and 50 mM sodium phosphate buffer, pH 8, containing 300 mM NaCl and 50 mM benzamidine (buffer A). The cell pellet was suspended in 25 mL of buffer A and cells were disrupted by sonication at 4°C. The cell lysate was centrifuged at 12000 ×g for 30 min at 4°C in a RC5 plus centrifuge, and the 6XHis-tagged recombinant proteins or protein complexes in the soluble fractions were purified as follows. The supernatant from the previous step was loaded on Ni-NTA agarose column preequilibrated with buffer A at 4°C. The column was washed extensively with buffer A, containing 25–50 mM imidazole, and the protein/protein complex was eluted with buffer A containing 300 mM imidazole. Fractions containing pure protein or protein complex were concentrated using centricon (Millipore PM10). Recombinant protein or protein complexes were dialyzed overnight against 100 volumes of 50 mM sodium phosphate buffer, pH 8, and the final preparations were stored at −20°C in the presence of 15% glycerol.

### 2.5. Endogenous Assay

To study the neutralizing effect of the immunity domain protein, *xcinA* gene was cloned with its native promoter along with immunity domain of *ximB* gene which gave rise to pXIM. This construct was transformed in *E. coli* DH5*α* to give rise to XIM strain. Empty pGEM-T Easy vector was considered as control and transformed in *E. coli* DH5*α* to give rise to GEM strain. Overnight grown strains GEM and XIM were subcultured in fresh medium and incubated till the OD_600_ reached 0.5. The cultures were diluted in fresh medium 1 : 100 and induced with 0.3 *μ*g mL^−1^ of mitomycin C (an inducer of xenocin native promoter). The optical densities of the cultures were monitored at 600 nm during different intervals.

### 2.6. Exogenous Toxicity Assay

The bacteriostatic activity of purified recombinant proteins/complexes were determined by the protocol as described earlier [13]. Briefly, LB agar plates without antibiotics were overlaid with 3 mL of soft nutrient agar containing indicator *E. coli *DH5*α* strain grown in M9 medium, and the protein complex was applied to sterile disks. The plates were incubated overnight at 37°C, and the sizes of clearance zones were recorded.

### 2.7. Hemolytic Activity Analysis

Freshly isolated rabbit blood cells were washed thrice with phosphate buffer saline (PBS) by centrifuging at 1000 ×g, 4°C for 10 minutes. Washed erythrocytes were resuspended in PBS to make a final concentration of 4%. The same volume (100 *μ*L) of protein (5 *μ*M) sample dissolved in PBS and erythrocytes suspension were added into wells of 96-well plate. PBS and water was used to establish 0 and 100% hemolysis, respectively. The plate was then incubated at 37°C for 1 hr and centrifuged at 1000 ×g for 5 minutes. The resulting supernatant was transferred to new wells, and the absorbance was determined at 540 nm on a continuous spectrum microtitre plate reader.

### 2.8. ATPase Assay with Immunity Protein and Its Domains

ATPase assay was performed with recombinant immunity protein and its domains. Protein samples of different concentrations were incubated with 0.2 *μ*Ci of [*γ*-^32^P] labelled ATP (6000 Ci/ mmol, PerkinElmer Life Sciences, USA) in a buffer containing 20 mM Tris-HCl (pH 8.0), 1 mM MgCl_2_, 100 mM KCl, 8 mM DTT, and 80 *μ*g/mL of BSA in a total reaction volume of 10 *μ*L. Samples were incubated at 37°C for 30 minutes. At the end of the reaction, 1 *μ*L of the reaction mixture was spotted on a polyethyleneimine thin-layer chromatography (TLC) plate (Sigma-Aldrich, USA) and air-dried. Chromatography was performed using 0.5 M LiCl and 1 M HCOOH as the running solvent. The TLC paper was air-dried and autoradiographed.

### 2.9. Circular Dichroism

The far-UV CD spectrum was recorded between 190 and 260 nm (500 *μ*L sample volume) on a Jasco J-810 spectropolarimeter equipped with a Jasco Peltier temperature controller at 25°C using 1 mm optical path length quartz cells and the step size was 0.5 nm with 1 nm bandwidth at a scan speed of 20 nm minute^−1^. Averages of 5 scans were obtained for blank and protein spectra, and the data was corrected for buffer contribution. Measurement was performed at protein concentration of 1 *μ*M under nitrogen flow. The secondary structure percentages were calculated using K2d computer modelling program. The results were expressed as mean residue ellipticity in units of degree/cm^2^/dmol^1^.

## 3. Results

**Figure 2:**
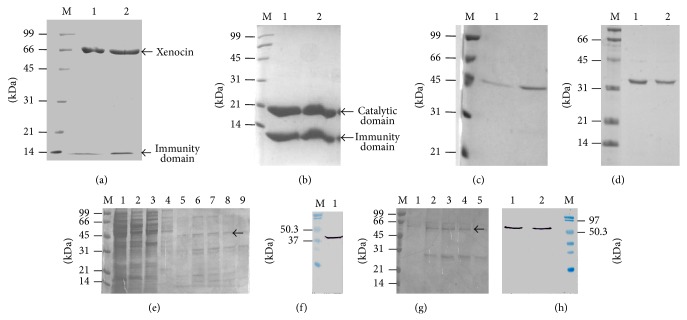
SDS-PAGE showing purification of recombinant proteins or protein complexes by Ni-NTA chromatography under native conditions. (a) Purification of xenocin-immunity domain (10 kDa) protein complex. Lane M: protein marker; lanes 1 and 2: purified fractions. (b) Purification of catalytic-immunity domain protein complex. Lane M: protein marker; lanes 1 and 2: purified fractions. (c) Purification of full length immunity protein (42 kDa). Lane M: protein marker; lanes 1 and 2: purified fractions. (d) Purification of hemolysin domain (32 kDa). Lane M: protein marker; lanes 1 and 2: purified fractions. (e) SDS-PAGE showing expression and purification of translocation domains of *xcinA *gene by Ni-NTA chromatography. Lane M: protein marker; lane 1: induced cells; lane 2: uninduced supernatant; lane 3: induced supernatant; lane 4: Wash 1; lanes 5 to 9: fraction number 2 to 6. Arrow indicates the purified translocation domain. (f) Western blot analysis of translocation domain with purified fraction using anti-His antibodies. Lane M, Prestained protein marker; Lane 1: purified sample. (g) SDS-PAGE showing the purification of translocation-receptor domains of *xcinA *gene by Ni-NTA chromatography. Lane M: protein marker; lanes 1 to 5: fraction number 3 to 7. Arrow indicates the expression and purification of translocation-receptor domain. (h) Western blot analysis of translocation-receptor domain with anti-His antibodies. Lane 1: induced cells; lane 2: Ni-NTA purified protein; Lane 3: prestained protein marker.

Phylogenetic analysis of the xenocin [14] and its immunity protein was done by preparing phylogenetic tree, using neighbour-joining method. Results showed that immunity protein formed a separate cluster in the very beginning as shown Figure 1(b). Attempt was made to express *xcinA* gene alone but transformants were not obtained. Further, *xcinA* gene was cloned along with N′ terminal immunity domain (10 kDa) of *ximB *gene. Purification of recombinant xenocin-immunity domain (10 kDa) protein complex from JC6 strain was done by Ni-NTA chromatography under native conditions. Two bands were visible in SDS-PAGE. One was at the position corresponding to 66 kDa and another below 14 kDa protein marker as shown in Figure 2(a) which corroborates with the size of xenocin and immunity domain of immunity protein, respectively. The yield of purified recombinant xenocin-immunity domain protein complex was 60 mg/L. Purification of recombinant catalytic-immunity domain protein complex with Ni-NTA chromatography under native conditions from JC4 strain [13] also showed two bands corresponding to the size of catalytic domain of *xcinA* and immunity domain of *ximB* as shown in Figure 2(b). The yield of purified recombinant catalytic-immunity domain protein complex was 100 mg/L. Purification of recombinant translocation domain of *xcinA* gene from JC8 strain with Ni-NTA chromatography showed multiple bands in SDS-PAGE as shown in Figure 2(e). Further, western blot was performed using purified fraction, probed with anti-His antibody, and the result showed single band at 38 kDa which corresponds to the size of recombinant translocation domain protein as shown in Figure 2(f). Purification of recombinant translocation-receptor domain protein with Ni-NTA chromatography from JC10 strain showed two prominent bands in SDS-PAGE. Upper band corresponded to ~52 kDa whereas lower band corresponded to ~28 kDa as shown in Figure 2(g). Western blot was performed using whole cell lysate and the purified fraction, probed with anti-His antibody, which showed a single band at 52 kDa corresponding to a size of recombinant translocation-receptor domain protein as shown in Figure 2(h). Purification of recombinant full length immunity protein, as well as its hemolysin domain protein from JC13 and JC14 strains, showed less but stable expression as shown in Figures 2(c) and 2(d), respectively. The yield of purified recombinant immunity protein and its hemolysin domain was 28 mg/L and 30 mg/L, respectively.

**Figure 3:**
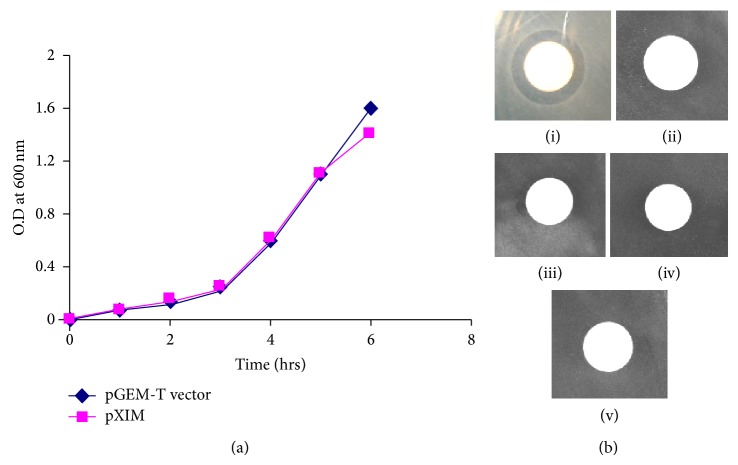
Neutralization of endogenous toxicity of xenocin expressed under its native promoter in the presence of mitomycin C. (a) Bacterial growth was monitored by determining the optical density at 600 nm; (■): XIM strain induced; (♦): GEM strain induced. (b) Exogenous toxicity assay using *E. coli* DH5*α* grown in M9 medium as target cells. (i) Purified recombinant xenocin-immunity domain protein complex; (ii) purified recombinant catalytic-immunity domain protein complex; (iii) purified recombinant immunity protein (42 kDa); (iv) purified recombinant hemolysin domain (32 kDa) of immunity protein; (v) buffer control.

Neutralization of endogenous toxicity of *xcinA* by immunity domain of *ximB* gene was determined by endogenous assay. Endogenous assay with XIM strain (harboring pXIM containing *xcinA* with its native promoter and first 85 amino acid residues of *ximB* gene) and GEM strain (harboring empty pGEM T-Easy vector) in the presence of mitomycin C showed the same growth profile as shown in Figure 3(a).

Exogenous toxicity assay was performed with purified recombinant xenocin-immunity domain (10 kDa) complex using *E. coli* DH5*α* as target cells. The zone of inhibition was observed as shown in Figure 3(b) (i). Purified catalytic-immunity domain protein complex was used to study for the bacteriostatic effect in the exogenous assays. The zone of inhibition was not observed in this case as shown in Figure 3(b) (ii). Similar results were observed when full length immunity protein (42 kDa) encoded by *ximB* gene or its hemolysin domain (32 kDa) was used for exogenous assay as shown in Figure 3(b) (iii) and (iv), respectively. Moreover, as expected zone of inhibition was not observed in buffer control experiment as shown in Figure 3(b) (v).

Protein-protein blast results (http://www.ncbi.nlm.nih.gov/BLAST/) of the immunity protein showed its similarity with hemolysin (AAF42109) and purine NTPase like protein (data not shown). Hemolytic assay with fresh rabbit red blood cells was performed with purified full length immunity protein as well as its immunity and hemolysin domain. Results showed that none of the protein had hemolytic activity (data not shown).

**Figure 4:**
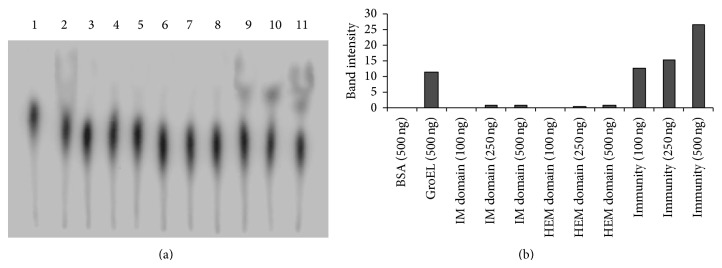
(a) ATPase assay with immunity domain (10 kDa), hemolysin domain (32 kDa), and full length immunity protein (42 kDa). (a) Lane 1: BSA (500 ng, negative control); lane 2: Gro EL (500 ng, positive control). Lane 3: immunity domain (100 ng); lane 4: (250 ng); and lane 5, (500 ng): Lane 6: hemolysin domain (100 ng); lane 7 (250 ng) and lane 8: (500 ng). Lane 9: immunity protein (100 ng); lane 10 (250 ng) and lane 11: (500 ng). (b) Histogram prepared by IMAGE J software on the basis of band intensity.

ATPase assay was performed with purified recombinant full length immunity protein, its immunity domain, and hemolysin domain. ATPase activity was not detected in recombinant immunity domain, hemolysin domain protein, and even in the purified BSA which was used as negative control as shown in Figure 4(a) lane 1. However, full length recombinant immunity protein (42 kDa) showed ATPase activity with increasing concentration of protein and was comparable to the ATPase activity of purified GroEL protein of *X. nematophila* which was used as the positive control, as shown in Figure 4(a) lane 2.

**Figure 5:**
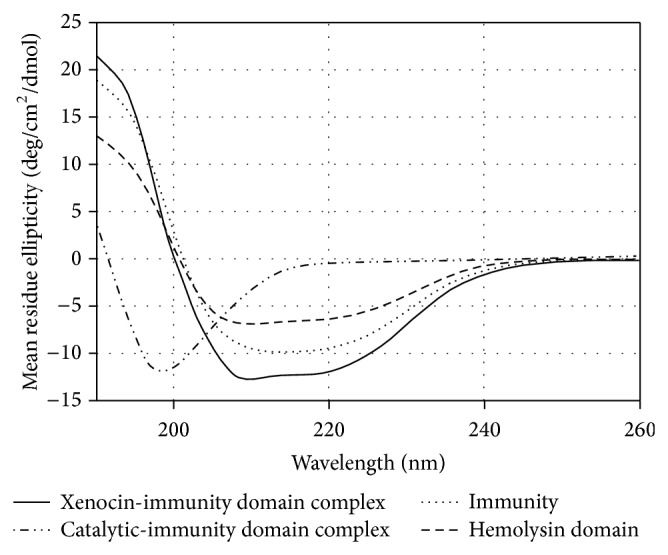
Far-UV CD spectra of purified recombinant xenocin-immunity domain (10 kDa) protein complex, catalytic-immunity domain protein complex, immunity protein, and its hemolysin domain.

The far UV spectra of purified recombinant xenocin-immunity domain (10 kDa) protein complex, catalytic-immunity domain protein complex, immunity, and its hemolysin domain were recorded at 25°C as shown in Figure 5. Recombinant xenocin-immunity domain protein complex was found to contain 41% *α*-helical structure and 21% *β*-sheet. Catalytic-immunity domain protein complex was found to contain 51% *β*-sheet and only 7% *α*-helical structure. In case of full length immunity protein 30% of the secondary structure was *α*-helical whereas 13% was *β*-sheet. Hemolysin domain of the immunity protein also had the same secondary conformation with 30% *α*-helical and 11% *β*-sheet content.

## 4. Discussion

Recombinant xenocin-(66 kDa-) immunity (42 kDa) protein complex has a broad range bacteriostatic property, inhibiting the growth of six insect gut resident bacterial species [13]. Due to only 30-31% primary sequence similarity with other bacteriocins, xenocin from the *X. nematophila* forms a distinct cluster in phylogenetic tree [14]. Phylogenetic analysis of immunity protein showed similar results, in which immunity protein from *X. nematophila* forms a separate cluster in the very beginning. This could be due to variable length of immunity protein from *X. nematophila*. Cognate immunity protein of xenocin consists of 368 amino acid residues and is a unique fusion of two different domains. Its N′ terminal (first 85 amino acid residues) showed similarity with immunity protein from other prokaryotic systems, whereas the C′ terminal showed similarity with hemolysin (*N. meningitidis* accession no. AAF42109) and purine NTPase like proteins.

Three-dimensional structure of xenocin has been recently deciphered by homology modelling in my lab [14]. It is a multidomain protein which consists of 576 amino acid residues. From its N′ terminal 1–327 amino acid residues form translocation domain (T), 328–476 amino acid residues form the middle receptor domain (R), and amino acid residues from 477–576 form the catalytic domain (C) at the C′ terminal [14].

While cloning *xcinA* gene alone in expression vector, not a single transformant was observed. One reason for this result could be the leaky expression of toxic *xcinA* gene. To address this question, *xcinA* gene was cloned along with N′ terminal immunity domain (10 kDa) of *ximB *gene. When the recombinant protein from JC6 strain was purified by Ni-NTA chromatography, xenocin was visible in SDS-PAGE at position corresponding to 66 kDa whereas immunity domain of immunity protein was observed below 14 kDa protein marker. This result showed that N′ terminal immunity domain (10 kDa) of immunity protein (42 kDa) encoded by *ximB* gene is enough to bind with and neutralize the *in vivo *toxic effect of *xcinA* gene. To confirm this result and to map the minimum functional domain of immunity protein required to abolish the *xcinA* gene toxicity *in vivo*, first 85 amino acids of immunity protein were cloned along with *xcinA* gene under its native promoter. Same growth profile of XIM strain (harboring pXIM) and GEM strain (harboring empty pGEM T-Easy vector) in the presence of mitomycin C confirmed that first 85 amino acid residues of *ximB* gene were able to neutralize the toxic activity of xenocin *in vivo*. Further, purification of recombinant catalytic-immunity domain protein complex with Ni-NTA chromatography under native conditions from JC4 strain [13] confirmed the minimal domains of *xcinA* and *ximB* genes that could be expressed and purified.

As we were unable to express *xcinA* gene alone which is composed of translocation, receptor, and catalytic domain an attempt had been made to clone, express, and purify the translocation domain alone or along with receptor domain of *xcinA *gene. In native conformation, translocation domain of bacteriocin like colicin E3 interacts with catalytic domain of E3 and immunity protein via receptor domain, and this interaction further provides stability to the translocation domain [15]. However, in recombinant translocation domain of *xcinA*, receptor and catalytic domains as well as immunity protein were missing which may be probably made it to attain an open conformation and be susceptible to proteases from the host cells. Hence, during the purification of translocation domain from pJC8, multiple bands were observed in SDS-PAGE. However, western blot with purified fraction when probed with anti-His antibody showed a single band corresponding to the size of translocation domain which confirmed the expression of translocation domain alone. Further, purification of recombinant translocation-receptor domain protein with Ni-NTA chromatography from JC10 strain, showed two prominent bands in SDS-PAGE rather than multiple bands. Upper band corresponded to ~52 kDa whereas lower band corresponded to ~28 kDa. western blot using whole cells and purified fraction when probed with anti-His antibody showed a single band at 52 kDa which is corresponding to a size of recombinant translocation-receptor domain protein. Therefore, we inferred that recombinant translocation domain along with receptor domain of the *xcinA* gene was more stable as compared to the recombinant translocation domain alone, but due to the absence of catalytic domain and immunity protein it was still prone to proteases of the host cell.

Endogenous toxicity assays with XIM and GEM strains were performed to identify the minimum domain required to neutralize the *xcinA* toxicity. Results showed that first 85 amino acids of the immunity protein were able to neutralize xenocin endogenous toxicity as both strains had the same growth profile. Moreover, from this experiment we inferred that expression of cognate immunity protein domain and its binding to the catalytic domain of xenocin occurs in the host cell simultaneously.

Receptor domain in bacteriocin plays a major role in the exogenous toxic effect, as it is the first domain which binds to ligand present on the surface of its target cells. Binding is followed by import of bacteriocin into the cells with the assistance of either Ton proteins (ExbB, ExbD, and TonB) or Tol proteins (TolA, -B, -Q, and -R) of periplasm and facilitate the translocation of the catalytic domain of bacteriocins into the periplasmic space of the target cell for further processing [5, 16–18]. Interaction of bacteriocins either with Ton or Tol proteins of periplasm occurs via Ton or Tol box present at their N′ terminal end of the translocation domain. Bacteriocins with RNase activity generally have Tol box in their translocation domain [4]. Interestingly, although xenocin has RNase activity, Tol box in its translocation domain has been replaced by Ton box [13]. This could be due to the domain swapping which generally occurs during the horizontal gene transfer in prokaryotes [4]. In the colicin E3-immunity complex, binding of the receptor domain of E3 to the surface protein on target cells leads to the conformational changes which assisted the immunity protein to dissociate from the protein complex [4, 18]. The immunity protein from *X. nematophila* is a unique fusion of immunity domain (10 kDa) and hemolysin domain (32 kDa) protein, and endogenous assay confirmed that immunity domain of *ximB* gene is enough to neutralize the detrimental effect of *xcinA* gene. Therefore, exogenous assay was performed with purified recombinant xenocin-immunity domain (10 kDa) complex using *E. coli* DH5*α* as target cells to determine translocational ability of xenocin-immunity domain (10 kDa) complex into the cytoplasm of target cells. Clear zone of inhibition was observed in the exogenous toxicity assay due to the detrimental effect of xenocin-immunity domain (10 kDa) protein complex. This result confirmed the functionality of translocation as well as receptor domain of xenocin and their individual roles in internalization of xenocin into the cytoplasm of target cells. Moreover, it also confirmed that hemolysin domain (32 kDa) of *ximB* gene has no role in the internalization of xenocin as well as in exogenous toxicity.

Catalytic domain of *xcinA *gene along with immunity domain of *ximB* gene was the minimum domain which could be expressed and purified. Therefore, purified catalytic-immunity domain protein complex was studied for the bacteriostatic effect in the exogenous assays. Results showed that zone of inhibition was not observed, which again confirmed the role of translocation as well as receptor domain of *xcinA* gene for its internalization into the cytoplasm on the target cells. Similar results were observed when full length immunity protein (42 kDa) encoded by *ximB* gene or its hemolysin domain (32 kDa) was used for exogenous assay. Moreover, as expected zone of inhibition was not observed in the experiment in which buffer was used as a negative control.

To neutralize the detrimental effect of bacteriocins, they are always expressed along with cognate immunity protein [3]. The molecular weight of the immunity protein in other prokaryotic systems is between 10 and 16 kDa; [4] however, immunity protein encoded by *ximB* gene corresponds to 42 kDa. Protein-protein blast using BLASTP (http://www.ncbi.nlm.nih.gov/BLAST/) of the immunity protein showed similarity with hemolysin (AAF42109) and purine NTPase like protein. Hemolytic assay with fresh rabbit red blood cells was performed with purified full length immunity protein as well as its immunity and hemolysin domains. Due to the partial primary amino acid sequence similarity with hemolysin protein, none of the protein/protein domains showed hemolytic activity.

As immunity protein also showed similarity with protein like purine NTPase's ATPase assay was performed with purified recombinant full length immunity protein, its immunity domain, and hemolysin domain. Although full length immunity protein showed ATPase activity, *in silico* analysis did not show Walker motif in its primary amino acid sequence, which is generally present in the proteins having ATPase activity [19]. The role of ATPase activity in the recombinant immunity protein is still not understood. Possibility of ATPase activity in immunity protein towards the secretion of recombinant xenocin-immunity protein complex cannot be ruled out at this stage as none of the proteins have any signal sequence at their either ends.

Secondary structure analysis of purified recombinant xenocin-immunity domain (10 kDa) protein complex, catalytic-immunity domain protein complex, immunity, and its hemolysin domain was done with far UV spectra. High percentage of *α*-helical structure of xenocin-immunity complex is attributed due to the helix turn helix structure of receptor domain of xenocin which corroborates with its recently deciphered three-dimensional structure [14]. Catalytic-immunity domain protein complex was found to contain 51% *β*-sheet and only 7% *α*-helical structure. This open conformation with high *β*-sheet might be beneficial to bind with its substrate (RNA) and act upon it [20, 21]. In case of full length immunity protein 30% of the secondary structure was *α*-helical where as 13% was *β*-sheet. Hemolysin domain of the immunity protein also had the same secondary conformation with 30% *α*-helical and 11% *β*-sheet content. Since immunity protein is a novel fusion of two different domains the role of its secondary is not deciphered yet.

## Figures and Tables

**Table 1:** Strains and plasmids used in this study.

Construct/strain	Characteristic	Source
*E. coli* DH5*α*	*supE44*Δ*lacU169 hsdR17 recA1 endA1 gyrA96 thi-1 relA1*Φ80 *dlacZ *ΔM15	Invitrogen
*E. coli* M 15	NaI^S^Str^S^RIF^S^Thi^−^Lac^−^Ara^+^Gal^+^MtI^−^F^−^RecA^+^Uvr^+^Lon^+^	Qiagen
*E. coli* BL21 (DE3) pLysS	F^−^ *omp*T *hsd*S_B_(*r*_*B*_^−^m_B_^−^) *gal dcm*(DE3)pLysS(Cm^R^)	Novagen
pGEM-T Easy	3 kb vector for cloning PCR fragments; Amp^r^	Promega
pQE30 and 31	3.4 kb expression vector; Amp^r^	Qiagen
pET 28 (a)	5.3 kb expression vector; kan^r^	Novagen
pJC2/JC2	pQE30 containing (*xcinA)* and *ximB* genes.	Ref. [13]
pJC4/JC4	pET28 containing catalytic domain and 270 bp partial *ximB *(N′ terminal 86 amino acids)	Ref. [13]
pJC5/JC5	pGEM-T Easy containing *xcinA* and *ximB* (255 bp) genes	This study
pJC6/JC6	pQE30 containing *xcinA* and *ximB* (255 bp) genes	This study
pJC7/JC7	pGEM-T Easy containing translocation domain of *xcinA* gene	This study
pJC8/JC8	pQE30 containing translocation domain of *xcinA* gene	This study
pJC9/JC9	pGEM-T Easy containing translocation-receptor domain of *xcinA* gene	This study
pJC10/JC10	pQE30 containing translocation-receptor domain of *xcinA* gene	This study
pJC11/JC11	pGEM-T Easy containing *ximB* gene	This study
pJC12/JC12	pGEM-T Easy containing hemolysin domain of *ximB* gene	This study
pJC13/JC13	pQE30 containing *ximB* gene	This study
pJC14/JC14	pQE31 containing hemolysin domain of *ximB* gene	This study
pXIM/XIM	pGEM-T Easy containing *xcinA* (with native promoter) and *ximB* (255 bp) genes	This study
pGEM/GEM	pGEM-T Easy in *E. coli* DH5*α*	This study
